# 613 Evaluation of Non-Euphoric Phytocannabinoid Elixir 14 (NEPE-14) Application in Deep Partial-Thickness Burn Wounds

**DOI:** 10.1093/jbcr/irac012.241

**Published:** 2022-03-23

**Authors:** David A Larson, Anders H Carlsson, Kristo Nuutila, Sean E Christy, Angela R Jockheck-Clark, Robert J Christy

**Affiliations:** US Army Institute of Surgical Research, San Antonio, Texas; The Metis Foundation, San Antonio, Texas; U.S. Army Institute of Surgical Research, San Antonio, Texas; U.S. Army Institute of Surgical Research, San Antonio, Texas; United States Army Institute of Surgical Research, Ft Sam Houston, Texas; U.S. Army Institute of Surgical Research, San Antonio, Texas

## Abstract

**Introduction:**

Combat-related burn wounds can be caused by exposure to a wide variety of agents including heat, electricity, radiation, chemicals, and friction. Early intervention can decrease injury severity by preventing excess inflammation and improve long term healing outcomes. In recent years, numerous studies have demonstrated that cannabinoids can trigger anti-inflammatory responses and promote wound closure. This study investigates whether a proprietary, non-euphoric, topical cannabinoid formulation (NEPE-14) facilitates burn wound healing when compared with Silverlon®, the military standard of care for burns incurred while in the field.

**Methods:**

Forty-eight deep-partial thickness burns were created on the dorsum of four anesthetized swine (*Sus scrofa domestica*) using a thermocoupled burn device at 100°C. One hour following burn, biopsies from each site were collected and either NEPE-14, NEPE-14 Vehicle Control, Silverlon®, or dry gauze was placed on the wound. Wounds were assessed on post-burn days (PBD) 3, 7 and 14. Assessments consisted of digital photographs, Laser-Speckle imagery (blood perfusion), FLIR imagery, MolecuLight® imagery and biopsies for histology and immunohistochemistry.

**Results:**

By PBD 14, no significant differences in re-epithelialization or contraction were observed between any of the tested treatment groups. Silverlon® had the highest percent re-epithelialization with 42%, and the Vehicle control has the lowest percent re-epithelialization with 28 %. Both NEPE-14 and dry gauze performed intermediately with 29% and 34% re-epithelialization, respectively. On PBD 14, the wounds of the NEPE-14 treated wounds contracted 8%, the vehicle control 7%, Silverlon® treated wounds 10%, and gauze treated wounds showed 9% contraction compared with the original wound area. Blood perfusion at PBD 14 indicated that NEPE-14 treated wounds had the lowest amount of observed blood flow and the gauze treated wounds contained the highest amount of observed blood flow. However, no statistically significant differences were observed.

**Conclusions:**

No statistically significant results were seen between treatment groups for contraction, re-epithelialization, or blood perfusion. Although not significant, blood perfusion results indicate that NEPE-14 treatment had the lowest amount of blood flow, which can be seen as an indicator of healthy underlying tissue and a wound re-epithelializing. At PBD 14, all treatment groups had slightly contracted after increasing in wound area on PBD 3 and 7, which is a pattern seen in similar burn injury studies. We are currently awaiting histological analysis to strengthen our re-epithelialization results and evaluate burn wound progression.

